# Multidrug Resistance: An Emerging Crisis

**DOI:** 10.1155/2014/541340

**Published:** 2014-07-16

**Authors:** Jyoti Tanwar, Shrayanee Das, Zeeshan Fatima, Saif Hameed

**Affiliations:** Amity Institute of Biotechnology, Amity University Haryana, Manesar, Gurgaon 122413, India

## Abstract

The resistance among various microbial species (infectious agents) to different antimicrobial drugs has emerged as a cause of public health threat all over the world at a terrifying rate. Due to the pacing advent of new resistance mechanisms and decrease in efficiency of treating common infectious diseases, it results in failure of microbial response to standard treatment, leading to prolonged illness, higher expenditures for health care, and an immense risk of death. Almost all the capable infecting agents (e.g., bacteria, fungi, virus, and parasite) have employed high levels of multidrug resistance (MDR) with enhanced morbidity and mortality; thus, they are referred to as “super bugs.” Although the development of MDR is a natural phenomenon, the inappropriate use of antimicrobial drugs, inadequate sanitary conditions, inappropriate food-handling, and poor infection prevention and control practices contribute to emergence of and encourage the further spread of MDR. Considering the significance of MDR, this paper, emphasizes the problems associated with MDR and the need to understand its significance and mechanisms to combat microbial infections.

## 1. Introduction

During the last few decades, the incidence of microbial infections has increased dramatically. Continuous deployment of antimicrobial drugs in treating infections has led to the emergence of resistance among the various strains of microorganisms. Multidrug resistance (MDR) is defined as insensitivity or resistance of a microorganism to the administered antimicrobial medicines (which are structurally unrelated and have different molecular targets) despite earlier sensitivity to it [1, 2]. According to WHO, these resistant microorganisms (like bacteria, fungi, viruses, and parasites) are able to combat attack by antimicrobial drugs, which leads to ineffective treatment resulting in persistence and spreading of infections. Although the development of MDR is a natural phenomenon, extensive rise in the number of immunocompromised conditions, like HIV-infection, diabetic patients, individuals who have undergone organ transplantation, and severe burn patients, makes the body an easy target for hospital acquired infectious diseases, thereby contributing to further spread of MDR. Studies from WHO report have shown very high rates of resistance (Table 1) in bacteria such as* Escherichia coli* against antibiotics as cephalosporin and fluoroquinolones,* Klebsiella pneumoniae* against cephalosporin and carbapenems,* Staphylococcus aureus* against methicillin,* Streptococcus pneumoniae* against penicillin, Nontyphoidal* Salmonella* against fluoroquinolones,* Shigella* species against fluoroquinolones,* Neisseria gonorrhoeae* against cephalosporin, and* Mycobacterium tuberculosis* against rifampicin, isoniazid, and fluoroquinolone causing common infections [3, 4] (like urinary tract infections, pneumonia, and bloodstream infections) and high percentage of hospital-acquired infections. A limited number of antifungal drugs are available for the treatment of chronic fungal infections. Resistance to drugs such as polyene macrolides (amphotericin B), azole derivatives (ketoconazole, fluconazole, itraconazole, and voriconazole), DNA and RNA synthesis inhibitors (flucytosine), and 1,3-*β*-glucan synthase inhibitors (echinocandins) exists in isolates of* Candida* spp.,* Aspergillus* spp.,* Cryptococcus neoformans*,* Trichosporon beigelii, Scopulariopsis* spp., and* Pseudallescheria boydii* [5]. Prolonged drug exposure and nonstop viral replication result in the advent of various resistant strains and persistence of infections despite therapy. This has made antiviral resistance a matter of concern in immunocompromised patients. Consequences of antiviral drug resistance were observed in immunosuppressed transplant recipients and oncology patients infected by either cytomegalovirus (CMV), herpes simplex virus (HSV),* Varicella-zoster* virus (VZV) [6], human immunodeficiency virus (HIV), influenza A virus, hepatitis C (HCV), or hepatitis B virus (HBV) [7]. Parasitic multidrug resistance has been analyzed in isolates of* Plasmodia, Leishmania, Entamoeba, Trichomonas vaginalis,* schistosomes [8, 9], and* Toxoplasma gondii* [10–12] against drugs such as, chloroquine, pyrimethamine, artemisinin, pentavalent antimonials, miltefosine, paromomycin, and amphotericin B [13, 14] as well as atovaquone and sulfadiazine. One of the most prime examples of disease prone to MDR is malaria, caused by* Plasmodium falciparum *[15]. Another protozoan parasite,* Entamoeba *spp., causes amoebiasis which is also a major public health threat in many tropical and subtropical countries [16]. A global health threat of schistosomiasis is also considered similar to that of malaria and other chronic diseases [9]. This review article emphasizes the significance of MDR, various mechanisms contributing to its development, and problems associated with MDR and its possible remedies.

## 2. Significance of MDR

Antimicrobial drugs have been used for several decades across the world. Surveillance in different regions of the world such as Africa, some parts of America, Eastern Mediterranean Region, Europe, South-East Asia, and Western Pacific Region has shown that many infectious microorganisms have evolved over the years and there is an alarming high number of antibiotic-resistant species enabling themselves to resist the inhibitory effects of these drugs. Not only a single but almost all the capable infecting agents (e.g., bacteria, fungi, virus, and parasite) have employed high levels of MDR with enhanced morbidity and mortality and, thus, are referred to as “super bugs.” Tuberculosis, pneumonia, HIV, influenza, malaria, yeast infections, and many other deadly diseases are major causes of deaths in modern era, therefore, indicating MDR as a serious worldwide threat to public health. The chances of controlling tuberculosis have decreased due to resistance of MTB to respective antibiotics, thus, making it a global concern. A 2012 survey suggests that an overall 6% of recent TB cases and 20% of formerly treated TB cases are likely to possess MDR, while 92 countries were found to have extensively drug resistant TB (XDR-TB). Another bacterial infection, pneumonia, has become untreatable because its causative agent has been found to be resistant to cephalosporin as well as carbapenems due to extended spectrum *β*-lactamases (ESBL) mediated mechanism [30], thereby rendering all available treatment using *β*-lactam antibiotics. In recent years, HIV drug resistance has driven the antiretroviral therapy failure to such an extent that it is charging exorbitant rates along with a number of side effects. The protozoan parasite responsible for malaria had embarked on showing resistance to some of its most effective drugs, chloroquine, artemisinin, and pyrimethamine [31]. This has resulted in replacement of these old ineffective drugs by novel drugs, which has increased the health care expenses. The emergence of resistance to antifungal drugs in invasive yeast infections, for example,* Candidiasis*, has led to worldwide morbidity and mortality, contributing to global economic burden. Antimicrobial resistance (AMR) or MDR is the reason why microbes fail to respond to standard drugs, thus, extending the duration of course of treatment further increasing the health care costs which tend to worsen the situation of people who are not capable of such expenses.

## 3. Problems Associated with MDR

**Figure 1:**
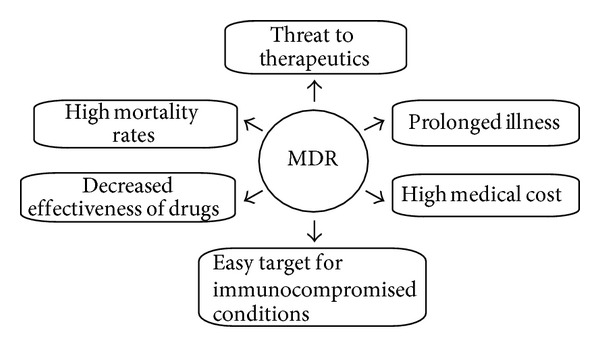
Problems associated with MDR.

Antimicrobial resistance is associated with high mortality rates and high medical costs and has a significant impact on the effectiveness of antimicrobial agents (Figure 1). MDR provokes obstruction in disease control by intensifying the possibility of spreading of resistant pathogens, thus, declining efficacy of treatment and, hence, resulting in prolonged time of infection in patient. The cost of treatment is also increased due to MDR as the pathogens have become resistant to commercially available drugs, which has triggered the use of more expensive therapies. The rate of success of present-day medical applications like organ transplantation and cancer chemotherapy has contributed immensely towards development of MDR. Differences in the resistance profiles of bacterial and fungal pathogens as well as the quality of public hygiene also have a considerable impact on the effectiveness of antimicrobial agents. Expansion of global trade and tourism lead to increased potential of MDR to spread all over the world and decrease in export and import of various products affecting the economy of developing countries [4, 32, 33].

## 4. Classification of MDR

**Figure 2:**
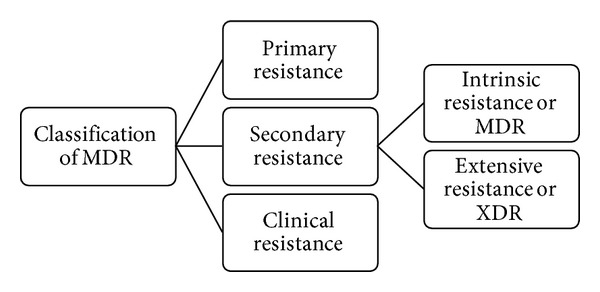
Classification of MDR.

Despite of administration of appropriate doses of drugs for a specific duration of time, survival of various microbial strains recommends the high levels of resistance developed in them. This clinical failure is due to not only the antimicrobial resistance but also the suppressed immune function, poor/deprived drug bioavailability, or increased rate of drug metabolism. Persistence of microbes after conventional/standard treatments points out different types of antimicrobial drug resistance which is an expanding problem in medical world. MDR can be classified (Figure 2) as primary or secondary resistance.

### 4.1. Primary Resistance

It occurs when the organism has never encountered the drug of interest in a particular host.

### 4.2. Secondary Resistance

Also known as “acquired resistance,” this term is used to describe the resistance that only arises in an organism after an exposure to the drug [5, 34]. It may further be classified as follows.

- *Intrinsic resistance:* it refers to the insensitivity of all microorganisms of a single species to certain common first-line drugs, which are used to treat diseases based on the clinical evidence of the patient. It is also known as multidrug resistance (MDR), for example,* Mycobacterium tuberculosis *to rifampicin and isoniazid or* Candida *spp. to fluconazole [5].
- *Extensive resistance:* it defines the ability of organisms to withstand the inhibitory effects of at least one or two most effective antimicrobial drugs. Also termed as XDR, this seemed to arise in patients after they have undergone a treatment with first line drugs, for example, XDR-TB resistance against fluoroquinolone [35, 36].

### 4.3. Clinical Resistance

In addition to the above-mentioned types, clinical resistance is defined by the situation in which the infecting organism is inhibited by a concentration of an antimicrobial agent that is associated with a high likelihood of therapeutic failure or reappearance of infections within an organism due to impaired host immune function. In other words, the pathogen is inhibited by an antimicrobial concentration that is higher than could be safely achieved with normal dosing [5].

## 5. Mechanisms of MDR

**Figure 3:**
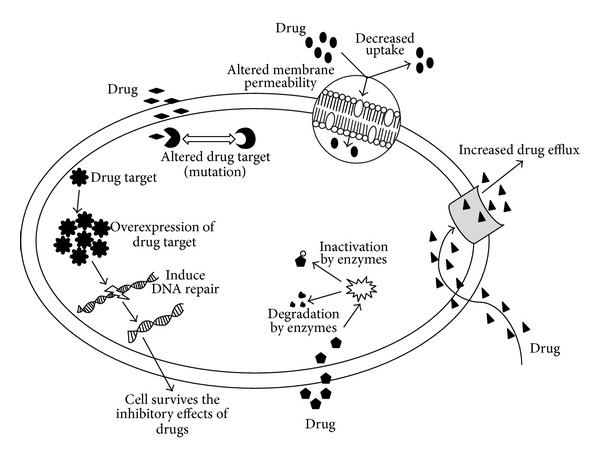
Mechanisms of MDR.

Resistance is the term referred to as the insensitivity of a microbe to an antimicrobial drug when compared with other isolates of the same species. Although several new drugs have been introduced commercially, this development of resistance among infectious microorganisms is increasing especially in patients under prolonged drug exposure [5]. Antimicrobial drugs generally act on the microbes either by inhibiting a metabolic pathway like nucleotide synthesis which in turn leads to the inhibition of DNA/RNA synthesis and further protein synthesis and disruption of the cell membrane or by competing with the substrate of any enzyme involved in cell wall synthesis (e.g., chitin synthase) [37]. Microorganisms have evolved a multitude of mechanisms to overcome the effectiveness of drugs, thereby surviving exposure to the drugs. This section will mainly describe the resistance mechanisms that the microbes develop to avoid getting killed by the drugs (Figure 3).

Cell wall, in both bacteria and fungi, plays a crucial role in their survival. As discussed above, drugs inhibit the cell wall synthesis by binding with the peptidoglycan layer in bacteria or affecting ergosterol synthesis (e.g., polyenes [38]) in fungi, thus, blocking the cell growth and division. These organisms undergo certain chromosomal mutations [39] or exchange of extrachromosomal DNA elements through conjugation or transformation (horizontal gene transfer) such as in* K. pneumoniae *[4], which can cause alteration in the cell membrane composition (e.g., a reduction in the ergosterol content in fungal plasma membrane) resulting in decreased permeability and uptake of drugs into the cell [1, 5, 37, 40]. Altered membrane composition (such as *β*-1,3-glucan and lipid content in fungal cell membrane) also leads to lack of active target sites for the drugs (e.g., echinocandins in fungi [38]) to bind. Mutations in the genes encoding for the target cause modifications at the molecular level and retain cellular function by reducing susceptibility to inhibition [1, 5, 37, 41].

Another mechanism of MDR was found to be an overexpression of drug target enzymes leading to target bypass due to modification in certain metabolic pathways (e.g., azoles and allylamines in fungi [38]), which causes production of alternate target molecules and interference in some protein synthesis. This can influence the access of drugs to the target sites.

Inactivation or enzymatic degradation of antimicrobials by hydrolysis of ester or amide bonds (such as resistance to *β*-lactams due to *β*-lactamases, etc.) and chemical transformation of these compounds by acetylation, phosphorylation, adenylation, glycosylation, and hydroxylation have also become increasingly apparent as causes of MDR [1, 37, 39]. The resistant strains of clinical isolates of different microorganisms have developed the ability to oxidize or reduce the antimicrobial compounds to prevent their interaction with the respective targets [37]. Antiviral drugs usually target viral DNA polymerase having the reverse transcriptase activity to inhibit the viral replication. Drug resistant mutant strains undergo mutations in the reverse transcriptase domains of the polymerase gene which affects the interaction between the drug and the enzyme. Resistance to the inhibitory effects of drug on the enzyme can also emerge due to any conformational changes or altered binding of substrate to the viral polymerase [6]. With the lack of effective antiparasitic vaccines yet in sight and new drugs developing slowly, MDR in parasites is emerging as a global public health threat. These parasites such as* Plasmodia *spp. and* Toxoplasma gondii*, like bacteria or fungi, also undergo certain point mutations/substitutions resulting in altered drug targets, alter calcium homeostasis in endoplasmic reticulum [11], and expel drugs (e.g., chloroquine, atovaquone, antifolate combination drugs, and artemisinin) out of the cells [10, 25].

MDR mediated by drug efflux pumps remains the predominant mechanism of MDR. The overexpression of genes encoding for ATP-binding cassette (ABC) transporter membrane proteins (e.g., P-glycoprotein (Pgp)), also known as the multidrug efflux pumps which are responsible for the export or expulsion of drugs out of the cell [3, 39, 42], usually generates MDR and continues cellular functions without any interference. Overexpression of P-glycoprotein, in* Entamoeba *spp. and* Leishmania *spp. membrane or multidrug resistant proteins (MRP), affects the fluidity and permeability, leading to an ATP-dependent efflux of the antimicrobials and decreasing their intracellular concentration [16, 43, 44]. MDR is also employed by cancer cells, which limits the long-term use of chemotherapy. An insight into the mechanisms involved in the chemoresistance, which can occur either at the beginning of the therapy (innate) or during the course of treatment, reveals that the cancer cells exhibit overexpression of certain multidrug resistance proteins (e.g., MRP and Pgp) which induce DNA repair mechanism, inhibit apoptosis, alter drug targets, and modify cell membrane composition as well as promoting an increased efflux of drugs preventing proper diffusion into the cells [45, 46].

## 6. Remedies for MDR

**Figure 4:**
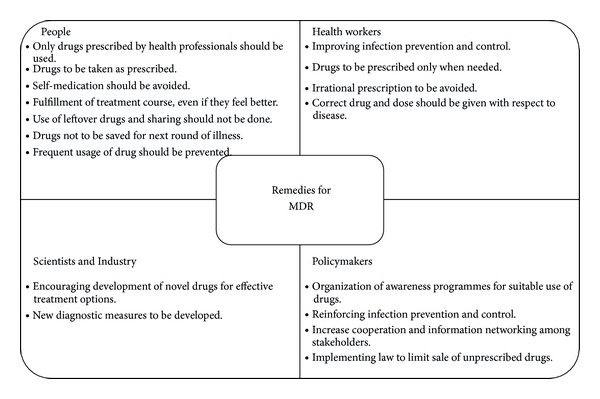
Remedies of MDR.

The development of MDR is a complicated issue which has become an international dreadful concern. To decrease the rise and spread of MDR, cooperative efforts (Figure 4) are requisite because diseases which were curable earlier are becoming major causes of deaths in this era [1, 4, 32]. Moreover, focusing on areas which are susceptible to inappropriate use of antimicrobials by implementation of antibiotic stewardship (defined as coordinated interventions designed to improve and measure the appropriate use of antimicrobials) is the need of the hour [47]. In fact, various antimicrobial stewardship programs (ASPs) are conducted nowadays to optimize antimicrobial therapy, reduce treatment-related cost, improve clinical outcomes and safety, and minimize or stabilize MDR [48]. Interventions through ASPs are either by restricting the availability of selected antimicrobial agents, known as “front-end,” or by examining broad spectrum use of antibiotics and then streamlining or discontinuing it, known as “back-end” [49]. Therefore, there is an urgent need of support and coordination at the global, regional, subregional, and national level to serve in future progress [4].

## 7. Conclusion

Rapid increase of severe systemic infections and the spread of resistant microorganisms are indisputable facts. Inadequacy of available antimicrobial drugs compels continuous development of newer drugs. Moreover, various awareness programmes which should facilitate their appropriate use to reestablish dominance over diseases must be implemented. MDR is an unavoidable natural phenomenon, posing a serious worldwide menace to public health. A cooperative action at global level is a must to combat the MDR. Pathogens tend to adopt various resistance mechanisms to survive the unfavorable conditions. Improved knowledge of molecular mechanisms controlling MDR should facilitate the development of novel therapies to combat these intransigent infections and will help cultivate a deeper understanding of the pathobiology of microbial organisms.

## Figures and Tables

**Table 1:** Common drug resistant microbes and diseases caused by them.

	Drug(s) resistant to	Typical diseases
Name of Bacterium		
*Escherichia coli *	Cephalosporins and fluoroquinolones	Urinary tract infections and blood stream infections
*Klebsiella pneumoniae *	Cephalosporins and carbapenems	Pneumonia, blood stream, and urinary tract infections
*Staphylococcus aureus*	Methicillin	Wound and blood stream infections
*Streptococcus pneumoniae*	Penicillin	Pneumonia, meningitis, and otitis
Nontyphoidal *Salmonella*	Fluoroquinolones	Foodborne diarrhoea, blood stream infections
*Shigella species*	Fluoroquinolones	Diarrhoea (bacillary dysentery)
*Neisseria gonorrhoeae*	Cephalosporins	Gonorrhoea
*Mycobacterium tuberculosis *	Rifampicin, isoniazid, and fluoroquinolone [4]	Tuberculosis
Name of Fungi		
*Candida* spp.	Fluconazole and echinocandins [5]	Candidiasis
*Cryptococcus neoformans*	Fluconazole [17]	Cryptococcosis
*Aspergillus *spp.	Azoles [18]	Aspergillosis
*Scopulariopsis *spp.	Amphotericin B, flucytosine, and azoles [19]	Onychomycosis
Name of Virus		
Cytomegalovirus (CMV)	Ganciclovir and foscarnet [20]	In AIDS and oncology patients
Herpes simplex virus (HSV)	Acyclovir, famciclovir, and valacyclovir [21]	Herpes simplex
Human immunodeficiency		
virus (HIV)	Antiretroviral drugs [22]	AIDS
Influenza virus	Adamantane derivatives (Amantadine and rimantadine) and neuraminidase inhibitors [23]	Influenza
*Varicella zoster* virus	Acyclovir and valacyclovir [21]	Chicken pox
Hepatitis B virus (HBV)	Lamivudine [24]	Hepatitis B
Name of Parasite		
*Plasmodia* spp.	Chloroquine, artemisinin, and atovaquone [25]	Malaria
*Leishmania* spp.	Pentavalent antimonials, miltefosine, paromomycin, and amphotericin B [13, 14]	Leishmaniasis
Schistosomes	Praziquantel and oxamniquine [26, 27]	Schistosomiasis
*Entamoeba*	Metronidazole [28]	Amoebiasis
*Trichomonas vaginalis*	Nitroimidazoles [29]	Trichomoniasis
*Toxoplasma gondii *	Artemisinin, atovaquone, and sulfadiazine [10–12]	Toxoplasmosis
